# Cerebellar Dural Arteriovenous Fistula Presenting with Hemorrhage: Diagnostic Imaging and Endovascular Management

**DOI:** 10.5334/jbsr.4035

**Published:** 2025-10-01

**Authors:** Christophe Sonck, Jef Huyskens

**Affiliations:** 1 Vrije universiteit Brussel, Health campus, Laarbeeklaan 103, 1090 Jette, Belgium; 2 Azorg ziekenhuizen, dienst radiologie, Merestraat 80, 9300 Aalst, Belgium

**Keywords:** cerebellum, dural arteriovenous fistula (dAVF), posterior fossa, intracranial hemorrhage, digital subtraction angiography (DSA), endovascular embolization, balloon-assisted technique, PHIL embolic agent

## Abstract

We report the case of a 45-year-old man with a rare cerebellar dural arteriovenous fistula (dAVF) presenting with dizziness and gait imbalance. MRI revealed a hemorrhagic lesion compressing the fourth ventricle. Digital subtraction angiography confirmed a Borden Type III dAVF, which was successfully treated by balloon-assisted embolization using PHIL 25%. This case draws attention to the importance of early diagnosis and intervention in posterior fossa dAVFs.

*Teaching point:* Cerebellar dAVFs, though rare, harbor a high risk of hemorrhage and require prompt imaging and endovascular treatment.

## Introduction

Dural arteriovenous fistulas (dAVFs) are abnormal shunts between meningeal arteries and dural venous sinuses or cortical veins. While relatively uncommon, dAVFs located in the posterior fossa are associated with a higher risk of hemorrhagic complications due to direct cortical venous drainage [1, 4]. Early diagnosis is essential as delayed treatment can result in permanent neurological deficits or death.

## Case Report

**Figure 1:**
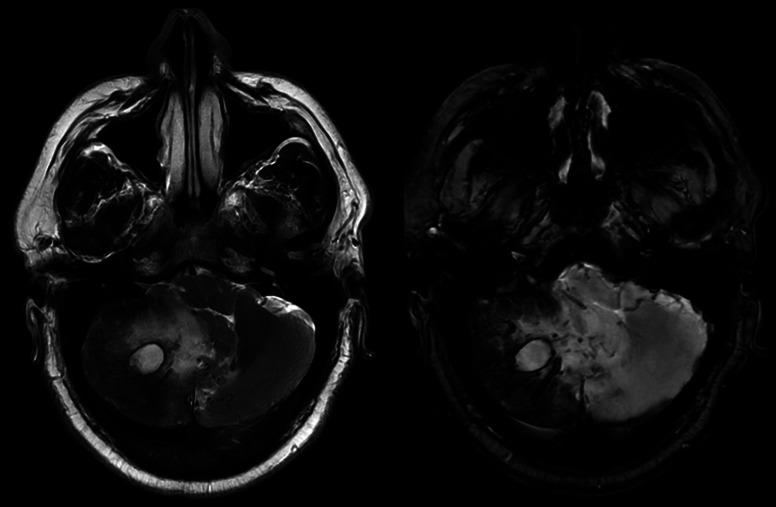
Axial magnetic resonance imaging of the cerebellum with T2-weighted images (left) and T2*-weighted images (right). On the left image, notice the edema surrounding the lesion in the right cerebellum with mass effect on the fourth ventricle. Visualization of hemosiderin (dark rim) due to prior hemorrhage on both, although more pronounced on the T2* sequence.

**Figure 2:**
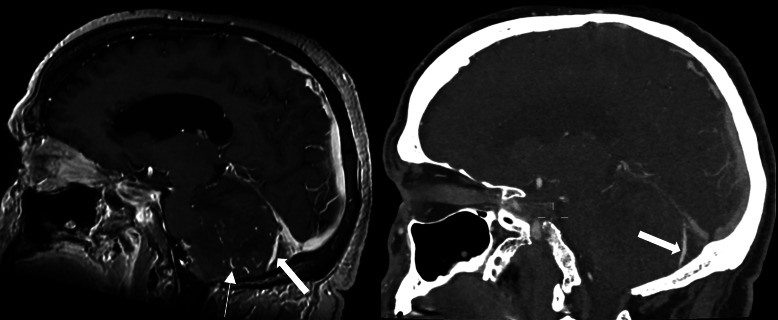
Sagittal magnetic resonance imaging of the brain with T1 images after contrast (left) and sagittal CT imaging of the brain after intravenous contrast (right). Notice the arterial collaterals in the cerebellum (thin arrow) and early enhancement of the inferior vermian vein (thick arrow) suggesting an arteriovenous fistula.

**Figure 3:**
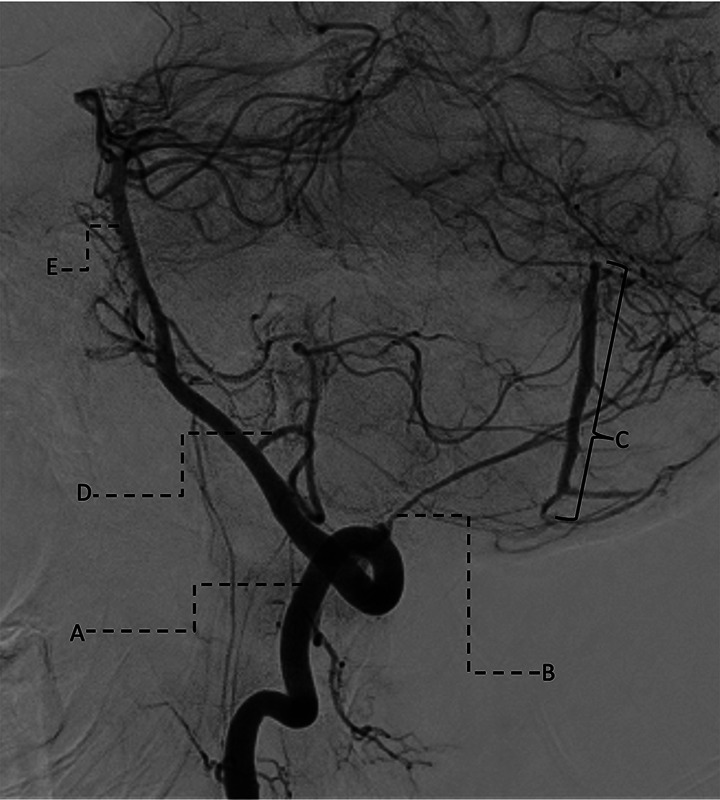
Pre-procedural DSA images of the AVF with enhancement of V3 segments of the right vertebral artery **(A)** and posterior meningeal artery **(B)** with drainage in the right inferior vermian vein **(C)**, confirming the dAVF. Additionally enhancing: posterior inferior cerebellar artery **(D)**, basilar artery **(E)**.

A 45-year-old male presented with progressive dizziness, unsteady gait, and horizontal nystagmus. Neurological examination showed impaired tandem walking without focal motor or sensory deficits. Contrast-enhanced brain MRI revealed a sharply demarcated intra-axial lesion (18 × 14 mm) in the right cerebellar hemisphere, hyperintense on T2-weighted images, with a surrounding hypointense rim suggestive of hemosiderin, indicating prior hemorrhage (Figure 1). Mass effect on the fourth ventricle raised concern for incipient hydrocephalus. CT angiography was highly suggestive of a dural arteriovenous fistula (Figure 2); therefore, a diagnostic catheter angiography was scheduled. The digital subtraction angiography (DSA) demonstrated a Borden Type III dAVF, with arterial supply from the V3 segment of the right vertebral artery and the posterior meningeal artery, draining into the right inferior vermian vein (Figure 3) [2, 3].

**Figure 4:**
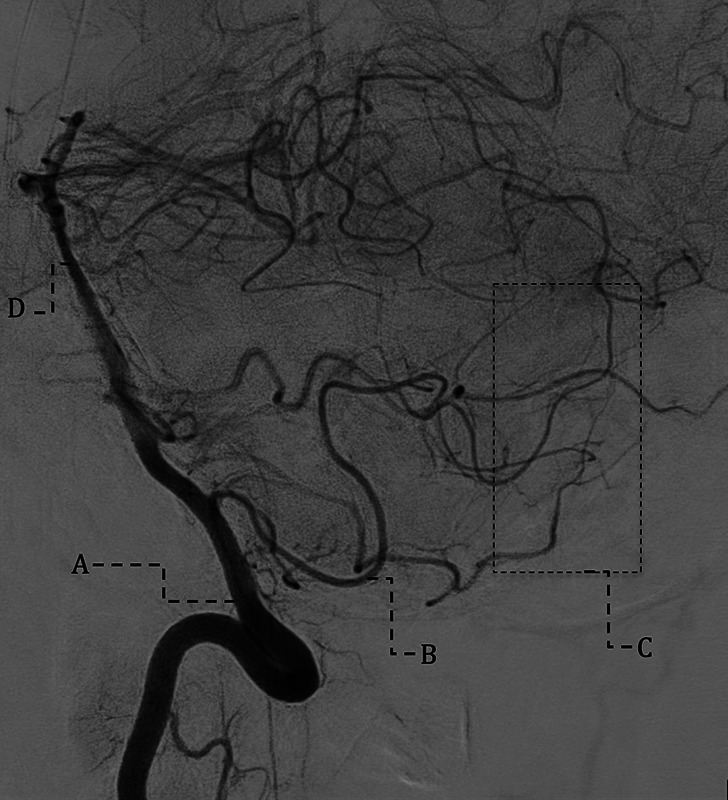
Six-month post-embolization DSA (right vertebral injection) shows normal opacification of the right vertebral artery **(A)**, posterior inferior cerebellar artery **(B)**, and basilar artery **(D)**. No residual filling of the treated dural arteriovenous fistula in region **C**, confirming complete occlusion.

Transarterial embolization was performed under general anesthesia. Access to the posterior meningeal artery was challenging, requiring use of a double-lumen balloon catheter. With the balloon inflated at the arterial ostium, 1.4 mL of PHIL 25% embolic agent was injected. Post-procedure angiography confirmed complete occlusion. An unenhanced CT 24 h post-procedure showed no complications. Prophylactic low-molecular-weight heparin was started, and a six-month angiographic follow-up was scheduled [4, 5]. This DSA (right vertebral injection) demonstrated normal opacification of the right vertebral artery, posterior inferior cerebellar artery, and basilar artery, with no residual or recurrent shunt, no early venous filling, and no cortical venous reflux—confirming durable cure (Figure 4).

## Discussion and Conclusion

Posterior fossa dAVFs with direct cortical venous drainage (Borden Type III) have a high hemorrhagic risk and often present with neurological symptoms such as gait disturbance or cranial nerve dysfunction [4]. MRI is useful to detect hemorrhage and mass effect, but DSA remains the gold standard for diagnosis and treatment planning [2, 3]. Balloon-assisted embolization with PHIL is a minimally invasive and effective treatment option, particularly in cases with tortuous access [5].

This case highlights the importance of early recognition of cerebellar dAVFs and demonstrates that successful treatment can be achieved through endovascular embolization. Rapid referral, appropriate imaging, and coordinated care are critical to improving patient outcomes.
